# Met-RANTES preserves the blood–brain barrier through inhibiting CCR1/SRC/Rac1 pathway after intracerebral hemorrhage in mice

**DOI:** 10.1186/s12987-022-00305-3

**Published:** 2022-01-21

**Authors:** Jun Yan, Weilin Xu, Cameron Lenahan, Lei Huang, Umut Ocak, Jing Wen, Gaigai Li, Wei He, Chensheng Le, John H. Zhang, Ligen Mo, Jiping Tang

**Affiliations:** 1 Department of Neurosurgery, Guangxi Medical University Cancer Hospital, Nanning, 530021 China; 2 Department of Physiology and Pharmacology, School of Medicine, Loma Linda University, Loma Linda, CA 92354 USA; 3 Department of Neurosurgery, School of Medicine, Second Affiliated Hospital, Zhejiang University, Hangzhou, 310009 China; 4 Department of Neurosurgery, School of Medicine, Loma Linda University, Loma Linda, CA 92354 USA; 5 Departments of Rheumatism, First Affiliated Hospital of Guangxi Medical University, Nanning, 530021 China; 6 Department of Anesthesiology, Loma Linda University, Loma Linda, CA 92354 USA

**Keywords:** Intracerebral hemorrhage, Blood–brain barrier, C–C chemokine receptor type 5, Met-RANTES

## Abstract

**Background:**

C–C chemokine receptor type 1 (CCR1) and its endogenous ligand, CCL5, participate in the pathogenesis of neuroinflammatory diseases. However, much remains unknown regarding CCL5/CCR1 signaling in blood–brain barrier (BBB) permeability after intracerebral hemorrhage (ICH).

**Methods:**

A total of 250 CD1 male mice were used and ICH was induced via autologous whole blood injection. Either Met-RANTES, a selective CCR1 antagonist, or Met-RANTES combined with a Rac1 CRISPR activator was administered to the mice 1 h after ICH. Post-ICH assessments included neurobehavioral tests, brain water content, BBB integrity, hematoma volume, Western blot, and immunofluorescence staining. The CCR1 ligand, rCCL5, and SRC CRISPR knockout in naïve mice were used to further elucidate detrimental CCL5/CCR1/SRC signaling.

**Results:**

Brain endogenous CCR1 and CCL5 were upregulated after ICH in mice with a peak at 24 h, and CCR1 was expressed in endothelial cells, astrocytes, and neurons. Met-R treatment reduced brain edema and neurobehavioral impairment, as well as preserved BBB integrity and tight junction protein expression in ICH mice. Met-R treatment decreased expression of p-SRC, Rac1, albumin, and MMP9, but increased claudin-5, occludin, and ZO-1 tight junction proteins after ICH. These effects were regressed using the Rac1 CRISPR activator. Administration of rCCL5 in naïve mice increased expression of p-SRC, Rac1, albumin, and MMP9, but decreased levels of claudin-5, occludin, and ZO-1 tight junction proteins. These effects in naïve mice were reversed with SRC CRISPR (KO).

**Conclusions:**

Our findings demonstrate that CCR5 inhibition by Met-R improves neurological deficits after ICH by preserving BBB integrity through inhibiting CCR1/SRC/Rac1 signaling pathway in mice. Thus, Met-R has therapeutic potential in the management of ICH patients.

**Supplementary Information:**

The online version contains supplementary material available at 10.1186/s12987-022-00305-3.

## Introduction

Spontaneous intracerebral hemorrhage (ICH) is a devastating neurovascular disease accounting for approximately 10%–15% of all strokes worldwide each year, and there is a profound lack of effective, available treatments [1]. If patients survive the primary brain injury, hematoma expansion may subsequently lead to life-threatening sequelae, such as the progressive exacerbation of cerebral edema, as well as neurological impairment, poor prognosis, and death [2]. Thrombin may initiate breakdown of the blood–brain barrier (BBB) and exacerbate perihematomal cerebral edema [3]. BBB disruption is an important component of pathogenesis in secondary brain injury (SBI) following ICH[4]. Decreased levels of endothelial tight junction proteins destroy BBB integrity, leading to angioedema and development of neurological dysfunction [5]. Previous studies have shown that protection of tight junction proteins can attenuate ICH-induced brain damage and improve the prognosis of neurological function [6, 7]. Therefore, strategies that target and preserve BBB integrity possess conceivable therapeutic potential for ICH patients.

Chemokines, a class of chemotactic cytokines, are named because of their essential role in leukocyte trafficking during inflammation. C–C chemokine receptor type 1(CCR1) is the first C–C chemokine receptor identified, and it belongs to G protein-coupled receptors. CCR1 is expressed by various cell types within the brain, including microvascular endothelial cells, vascular smooth muscle cells, astrocytes, and neurons [8–10]. In the ischemic stroke model, CCR1 immunoreactivity was predominantly localized to the cerebral endothelium and ependyma [8]. CCR1 activation contributes to the BBB damage associated with a variety of neuroinflammatory diseases, including multiple sclerosis (MS), diabetes mellitus, and Alzheimer’s disease [11, 12]. RANTES (CCL5), an endogenous ligand of CCR1, has pro-inflammatory effects in animal models of cerebral ischemia, Alzheimer’s disease, and autoimmune encephalomyelitis (EAE) [13–15]. Met-RANTES (Met-R), an N-terminally modified human RANTES, is a selective CCR1 antagonist that inhibits the agonistic activity of CCR1 [16]. Met-R was effective in treating immune-mediated acute and chronic models of tissue inflammation in vivo.[17, 18]. However, the effects of CCR1 antagonism on post-ICH BBB integrity have not been elucidated.

SRC belongs to the SRC kinase family, a group of membrane-associated, non-receptor tyrosine kinases that have been implicated in several signaling pathways, suggesting a broad spectrum of cell physiology [19]. SRC also has a critical function in which it facilitates epithelial cell adhesion and motility [20]. SRC regulates cell migration and invasion in breast cancer metastasis by stimulating the FAK/SRC/Rac1 signaling pathway [21]. Rac1, also known as Ras-related C3 botulinum toxin substrate 1, is a pleiotropic governor of several cellular processes, such as the cell cycle, adhesion, migration, invasion, and mesenchymal transition (EMT) [22]. Recent studies have shown that the chemokine receptor, CCR7, initiates an endomembranal signaling complex for spatial Rac1 activation through the SRC/RAC1 signaling pathway in dendritic cells [23].

Collectively, we hypothesized that inhibition of CCR1 with Met-R could protect BBB damage and improve neurological deficits after ICH in mice. Furthermore, this present study explored the mechanism by which Met-R provided BBB protection, and found that it functions through inhibiting CCR1/SRC/RAC1 pathway.

## Materials and methods

### Animals

All experimental protocols in this research were approved by the Institutional Animal Care and Use Committee (IACUC) at Guangxi Medical University. All procedures of animal experiments are reported in according to the ARRIVE (Animal Research: Reporting of In Vivo Experiments) guidelines. All animal care and use were conducted in accordance with Health’s Guide for the Care and Use of Laboratory Animals for the National Institutes. A total of 250 male CD1 mice (2–3 months, weight about 30 g–40 g) were used in this study. The animals were housed in a temperature-controlled room on a 12-h light–dark cycle, with ad libitum access to food and water.

### Experimental design

**Fig. 1:**
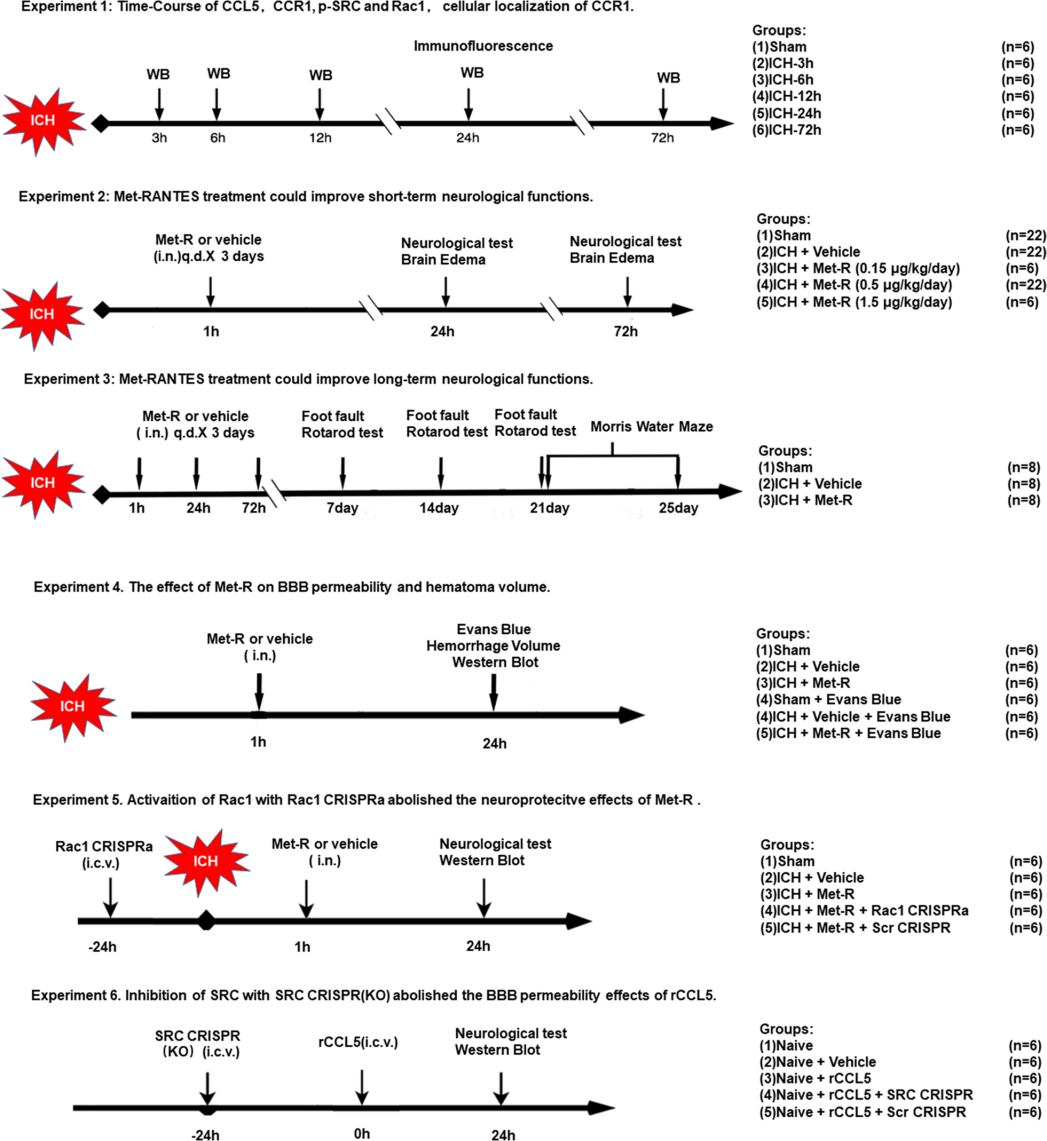
Experimental design of animal study. Experiment 1: Time course study and cellular localization. Experiment 2: Effects of Met-R on short-term outcomes including neurobehavior and brain edema at 24 and 72 h after ICH. Experiment 3: Long-term outcome study up to 25 days after ICH. Experiment 4: BBB permeability and hematoma volume at 24 after ICH. Experiment 5: Mechanism study to elucidate the role of Rac1 as a downstream mediator of CCR1. Experiment 6: Mechanism study to elucidate the rCCL5-mediated activation of CCR1 in naïve animals. ICH: intracerebral hemorrhage; Met-R: Met-RANTES; CCR1: C–C chemokine receptor type 1; rCCL5: recombinant chemokine ligand 5; WB: Western blot; IF: immunofluorescence staining; i.c.v.: intracerebroventricular

In our study, the animals were randomized into five main experiments using ICH mice and naïve mice. The experimental design with five separate experiments and timelines are shown in Fig. 1. The amount of mice and their respective mortality rates are reported in Additional file 1: Table S1. The animals were randomly placed into different trial groups. All group information was blinded to the researchers who performed experiments and analyzed research data.

Experiment I. Western blots were conducted to assess the endogenous brain expressions of CCR1, CCL5, p-SRC, and Rac1 using specimens procured from the right hemisphere of each group. Animals were randomly divided into 6 groups: sham (n = 6), ICH 3 h (n = 6), ICH 6 h (n = 6), ICH 12 h (n = 6), ICH 24 h (n = 6), and ICH 72 h (n = 6). An additional 2 mice were used for immunofluorescence staining at 24 h post-ICH. Cellular co-localization of CCR1 was assessed by double-labeling immunofluorescence labeling to co-localize CCR1 with endothelial cells (vWF), glial fibrillary acidic protein (GFAP), and neuronal specific nuclear protein (NeuN) at 24 h post-ICH.

Experiment II. To determine the optimal treatment dose and assess the protective effects on the BBB from intranasal administration of CCR1 selective antagonist, Met-R, at 1 h after ICH. Thirty animals were randomly assigned into five groups: sham (n = 6), ICH + vehicle (n = 6), ICH + Met-R (0.15 μg/kg/day, n = 6), ICH + Met-R (0.5 μg/kg/day, n = 6), and ICH + Met-R (1.5 μg/kg/day, n = 6). Brain water content (BWC) and neurologic outcomes were evaluated at 24 h post-ICH. Based on BWC results and neurologic outcomes, the medium dose (0.5 μg/kg/day) of Met-R was selected as the optimal dose for further investigation. The effects of Met-R on BWC and the neurologic outcomes were further accessed at 72 h post-ICH. A total of eighteen mice were randomly divided into three groups: sham (n = 6), ICH + vehicle (n = 6), and ICH + Met-R (0.5 μg/kg/day, n = 6). To evaluate Evans blue (EB) extravasation and hemorrhagic volume at 24 h post-ICH, another set of eighteen mice were randomized to sham (n = 6), ICH + vehicle (n = 6), and ICH + Met-R (0.5 μg/kg/day, n = 6) groups.

Experiment III. Twenty-four animals were randomized into three groups: sham (n = 8), ICH + vehicle (n = 8), and ICH + Met-R (0.5 μg/kg/day, n = 8) to evaluate long-term neurological outcomes. Met-R at a dose of 0.5 μg/kg was administered daily for 3 consecutive days. Foot fault and rotarod tests were conducted on days 7, 14, and 21 post-ICH. Morris water maze was performed on days 21–25 post-ICH.

Experiment IV. To verify the potential blood–brain barrier preservation mechanisms of Met-R after ICH, the selective Rac1 CRISPR activator (Rac1 CRISPRa) or Scramble CRISPR (Scr CRISPR) was delivered intracerebroventricularly (i.c.v) 24 h pre-ICH, and the specific CCR1 antagonist, Met-R (0.5 μg/kg/day), was administered intranasally 1 h post-ICH induction. Thirty mice were randomly distributed into five groups (n = 6/group): sham, ICH + vehicle (PBS), ICH + Met-R (0.5 μg/kg/day), ICH + Met-R + Scr CRISPR, and ICH + Met-R + Rac1 CRISPRa.. Neurobehavioral tests and western blots were completed 24 h post-ICH.

Experiment V. To further examine the detrimental effects of CCR1/ SRC activation, rCCL5, SRC CRISPR knockout, and Scramble CRISPR were administered via i.c.v. injection in naïve mice. Thirty mice were randomized into five groups (n = 6/group): sham, sham + vehicle, Naïve + rCCL5, Naïve + rCCL5 + Scr CRISPR, and Naïve + rCCL5 + SRC CRISPR (KO). Neurobehavioral tests and western blots were performed 24 h post-rCCL5 injection.

### Intracerebral hemorrhage mouse model

Autologous whole blood (30 μl) injection was used to induce ICH as previously described [24, 25]. Briefly, the mice were anesthetized using intraperitoneal injection of a mixture of ketamine (100 mg/kg) and xylazine (10 mg/kg). They were positioned on a stereotactic head frame (Kopf Instruments, Tujunga, CA, USA) and a Chlortetracycline Hydrochloride Eye Ointment (Beijing Shuangji Pharmaceutical Co., Ltd.) was applied to prevent the eyes from drying during surgery. Autologous whole blood was transferred into a 27-gauge needle on a 250 μl Hamilton syringe through a nonheparinized capillary tube. A 1-mm cranial burr hole was drilled and the needle of a Hamilton syringe was inserted at the following coordinates relative to the bregma: 0.2 mm posterior, 2.2 mm lateral, and 3.0 mm below the dura. To limit the backflow of blood along the needle track, two-stage of injection was performed. At first, a total of 5 μL of blood was injected into the right basal ganglia at a rate of 2 μL/min using a microinjection pump. After 5 min, another 25 μL of blood was injected at the same rate at a depth of 3.5 mm dorsoventrally, resulting in a total injection volume of 30 μl. Upon completion of blood injection, the needle remained in place for an additional 5 min to avoid possible leakage. After which, it was withdrawn slowly at a rate of 1 mm/min. Bone wax was used to seal the cranial burr hole and the scalp was subsequently sutured. Lastly, 0.4 μl of normal saline was administered subcutaneously to prevent postsurgical dehydration. Under careful monitoring, the mice were allowed to fully recover in a recovery cage on a warm heating pad. Sham mice underwent an identical procedure without blood injection.

### Drug administration

Met-R was procured from R&D Systems and was dissolved in a PBS solvent prior to injection. Three different doses of Met-R (0.15 μg/kg/day, 0.5 μg/kg/day, and 1.5 μg/kg/day) were assessed, which were intranasally delivered to the mice in ICH + Met-R group at 1 h post-surgery [26]. Rac1 CRISPRa (Santa Cruz Biotechnology, INC.), a specific Rac1 activator, was delivered i.c.v 24 h before ICH [27]. Recombinant CCL5 (rCCL5) (R&D Systems) was dissolved in a PBS solvent and administered i.c.v in Naïve mice as previously reported [28]. SRC CRISPR knockout or scramble CRISPR (scr CRISPR) (Santa Cruz Biotechnology, Dallas, TX, USA.) was administered i.c.v. at a rate of 1 μL/min using a microinjection pump [27].

### Intracerebroventricular injection

Intracerebroventricular injection was performed as previously reported [29]. Briefly, the mice were anesthetized using an intraperitoneal injection of a mixture of ketamine (100 mg/kg) and xylazine (10 mg/kg). A 1-mm cranial burr hole was drilled on the skull using stereotactic methods at coordinates of 0.22 mm posterior and 1.0 mm lateral from bregma: A 27-gauge needle of a Hamilton syringe was inserted into the left lateral ventricle at a depth of 2.25 mm below dura through the cranial burr hole. The rCCL5 or CRISPR was delivered at a rate of 0.667 μL/min using a microinjection pump. Upon completion of injection, the needle remained for 5 min. After which, it was withdrawn at a rate of 1 mm/min. Finally, bone wax was used to seal the cranial burr hole.

### Assessments of BBB permeability and hematoma volume

Evans blue dye extravasation assay was used to evaluate BBB permeability [30]. Evans Blue dye (2%, 0.25 ml, E2129; Sigma-Aldrich) was administered through intraperitoneal injection 4 h prior to sacrificing animals. Under deep anesthesia from isoflurane, mice were transcardially perfused with PBS. Brains were quickly harvested and then separated into right and left hemispheres. All brain tissues were snap-frozen in liquid nitrogen and stored at − 80 °C until used. Brain samples were homogenized using PBS (1 ml/300 g), followed by sonication. After being centrifuged at 15,000 rpm for 30 min, the supernatant was incubated overnight at 4 °C with an equal volume of trichloroacetic acid (50%). The next day, the samples were centrifuged using the same parameters. Evans blue dye extravasation was measured using a spectrophotometer (620 nm; Genesis 10uv; Thermo Fisher Scientific, Waltham, MA), quantified using a standard curve, and normalized to tissue weight.

Hematoma volume was assessed by spectrophotometric measurements [31, 32]. Frozen whole brain tissue was homogenized in PBS for 30 s (Tissue Miser Homogenizer; Fisher Scientific, Pittsburgh, PA), and sonication on ice with a pulse ultrasonication for 1 min to lyse the erythrocytic membranes. The sample was centrifuged at 13,000 rpm for 30 min, followed by supernatant collection. Drabkin’s reagent (80 μl, Sigma-Aldrich) was added to supernatant and was left to react for 15 min. Absorbance measurements were performed using a spectrophotometer (540 nm; Genesis 10uv; Thermo Fisher Scientific, Waltham, MA). The value of absorbance was converted into a hemorrhagic volume (μL) based on a standard curve [32].

### Short-term neurological outcome assessment

Short-term neurological outcomes were accessed at 24 h and 72 h post-ICH by an investigator blinded to group information. Modified Garcia (mGarcia), forelimb placement, and corner turn tests were used as previously reported [33]. Modified Garcia scores were evaluated by 7 subtests including spontaneous activity, proprioception, vibrissae, symmetry of limb movement, turning, forepaw outstretching, and climbing. The total test scores ranged from 3 to 21, with each subtest score ranging from 0 to 3. Forelimb placement test was utilized to assess the responsiveness of vibrissae stimulation in mice, and the results were documented as the percentage of successful left forelimb placements out of 10 successive stimulations. The following formula was used to calculate left forelimb placement: left forelimb placement/(left forelimb placement + right forelimb placement) × 100%. During the forelimb placement test, the vibrissa was stimulated, and the countertop placement of the ipsilateral forelimb was documented. In the corner turn test, the mice were permitted to advance into a 30° angle corner. After which, the mice could exit the corner by turning either right or left. The percentages of left turns in 10 trials were calculated.

### Long-term neurological performance

Foot-fault and rotarod tests were conducted to evaluate sensorimotor coordination and balance weekly during weeks 1–3 after ICH. The Morris water maze test was completed on days 21–25 after ICH to assess memory and spatial learning capacity as previously described [34].

### Brain water content

Brain water content (BWC) was assessed at 24 h and 72 h post-ICH using the wet/dry method as previously reported [35]. Under deep anesthesia, the mice were decapitated. The brains were quickly harvested and separated into ipsilateral and contralateral cortices, basal ganglia, and cerebellum. Each segment was weighed using an electric analytic balance to obtain the wet weight, followed by incubation in 100 °C oven for 24 h to procure the dry weight. The following formula was used to calculate BWC: brain water content (%) = [(wet weight − dry weight)/wet weight] × 100%.

### Western blot assay

Western blotting assay was performed as previously reported [36]. The ipsilateral/right brain hemispheres were homogenized in RIPA lysis buffer for protein extraction, and supernatants were collected after centrifugation at 4 °C for 30 min at 14,000*g*. Equal amounts of protein samples were loaded onto an SDS-PAGE gel. The samples underwent electrophoresis and were transferred to a nitrocellulose membrane. The membrane was blocked and incubated overnight at 4 °C using the following primary antibodies: rabbit anti-CCR1 (1:1000, PA1-41062), mouse anti-CCL5 (1:1000, sc-373984), rabbit anti-SRC (1:1000, ab47405), rabbit anti-p-SRC (1:1000, ab40660), mouse anti-Rac1 (1:1000, ab33186), rabbit anti-albumin (1:1000, ab207327), rabbit anti-MMP9 (1:1000, ab38898), mouse anti-claudin-5 (1:1000, sc-374221), mouse anti-occludin (1:1000, sc-133256), rabbit anti-ZO-1 (1:1000, sc-10804), and mouse anti-β-actin (1:2000, sc-47778). The following day, the membranes were incubated with their respective secondary antibodies (1:3000, Santa Cruz) at room temperature for 2 h. An ECL reagent (AmershamBiosciences, Arlington Heights, PA) was used to probe the blot bands, which were then visualized using an image system (Bio-Rad, Versa Doc, model 4000). The protein immunoblot images were analyzed using ImageJ software (ImageJ 1.4, National Institutes of Health, USA).

### Double immunofluorescence staining

Double immunofluorescence staining was performed as previously reported [37]. In each animal, three 8-μm thick coronal brain sections at the level of basal ganglion (0.48 mm post to bregma) were stained. For each slice, we analyzed 4 fields of view (FOV) around the perihematomal area clockwise. The results were averaged from 4 (FOVs)/slice of 3 slices/animal for histological or immunohistochemical assessments. Under deep anesthesia, the animals were transcardially perfused with cold PBS and 10% formalin 24 h post-ICH. The brain samples were harvested, post-fixed in 10% formalin at 4 °C for 24 h and dehydrated for an additional 3 days in the 30% sucrose solution. The brain tissues were embedded in OCT and then cut into 10-μm thick slices using cryostat (CM3050S, Leica Microsystems, CITY, USA).The primary antibodies (Abcam, MA, USA) were used as follows: rabbit anti-CCR1 (1:200, PA1-41062), rabbit anti-GFAP (1:200, ab16997), rabbit anti-NeuN (1:200, ab177487) and rabbit anti-vWF (1:200, ab6994). Lastly, the slices were incubated with fluorescence-conjugated secondary antibodies (1:500, Jackson ImmunoResearch, PA, USA) at 25 °C for 2 h. Co-localizations were visualized and photographed using a fluorescent microscope (Olympus OX51, Tokyo, Japan).

### Statistical analysis

Statistical analysis was conducted using SPSS 24.0 (IBM Corp., USA). Data were demonstrated as mean ± SD, unless otherwise stated. One-way ANOVA was performed for comparison between different groups (> 2 groups) followed by post-hoc test, including Bonferroni correction for multiple comparisons. For non-normal distributed values, ANOVA for nonparametric values (Kruskal–Wallis test) was used followed by correction for multiple comparisons with the Dunn method. A Mann–Whitney U test was used for individual comparison of independent values within a group. Additionally, two-way repeated-measures ANOVA was applied to analyze the data of long-term water maze test. P < 0.05 was considered statistically significant.

## Results

### Mortality and exclusion

A total of 250 animals were used for this study. Of those, 56 were sham, 30 were naïve, and 164 animals underwent ICH induction. None of the mice in the sham or naïve groups died in this study. The overall ICH mortality in the whole study was 4.8% (12/250) in the absence of statistical differences found among experimental groups. No animals were excluded from this study (Additional file 1: Table S1).

### Endogenous brain expression of CCL5, CCR1, p-SRC, and Rac1 increased after ICH

**Fig. 2:**
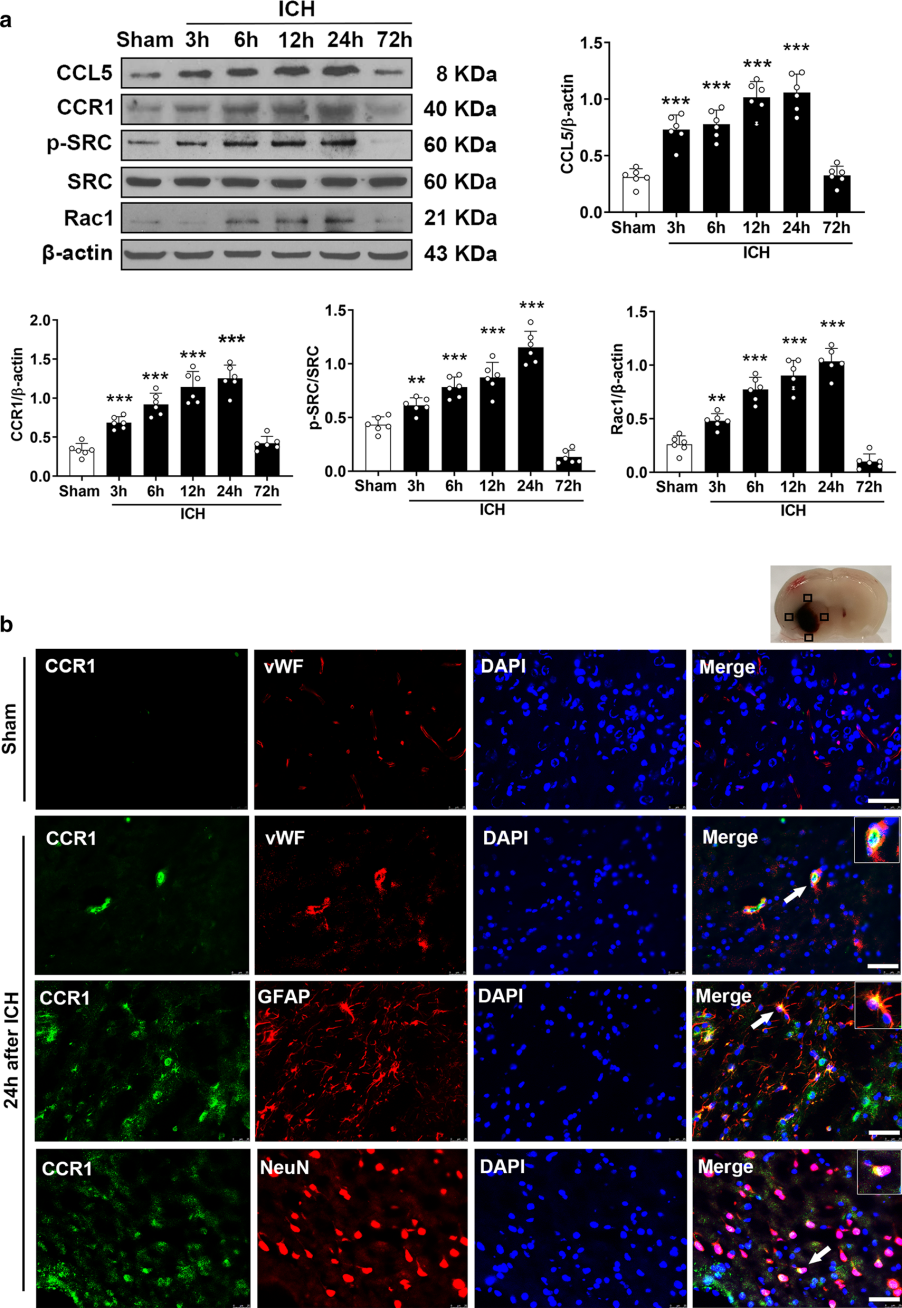
Temporal expressions of CCR1, CCL5, p-SRC, and Rac1 after ICH in mice. **A** Representative western blot images and quantitative analyses of time course of CCR1, CCL5, p-SRC, and Rac1 expressions within the ipsilateral hemisphere after ICH. Data were expressed as mean ± SD. ^*^p < 0.05, ^**^p < 0.01, ^***^p < 0.001 vs. sham group. n = 6/group. **B** The four black squares indicated perihematomal regions for microphotograph of the immunofluorescence staining. Double immunofluorescence staining for CCR1 (green) in endothelial cells (vWF, red), astrocytes (GFAP, red), and neurons (NeuN, red) within peri-hematoma area 24 h after ICH. Nuclei were stained with DAPI (blue). Scale bar = 50 μm. n = 2/group

Endogenous expression of CCL5, CCR1, p-SRC, and Rac1 within ipsilateral (right) brain hemisphere was assessed via western blot at 3, 6, 12, 24, and 72 h after ICH. A significant increase in CCL5 levels occurred 3 h post-ICH and peaked at 24 h post-ICH when compared to the sham group. CCR1 expression was significantly increased 6–24 h post-ICH compared to sham, but the expression levels were decreased 72 h post-ICH. There was a marked increase of endogenous p-SRC levels observed at 6 h, which was maintained at a relatively high level at 24 h. Rac1 expression was significantly elevated 3–24 h post-ICH compared to the sham group. (*P* < 0.05; Fig. 2a). Interestingly, double immunostaining of CCR1 with vWF (endothelial cell marker), GFAP (astrocyte marker), and NeuN (neuron marker) revealed that the increased CCR1 expression was co-localized with endothelial cells, astrocytes, and neurons at 24 h post-ICH (Fig. 2b).

### Met-R treatment ameliorated ICH-induced neurobehavioral dysfunction and reduced brain edema at 24 and 72 h after ICH

**Fig. 3:**
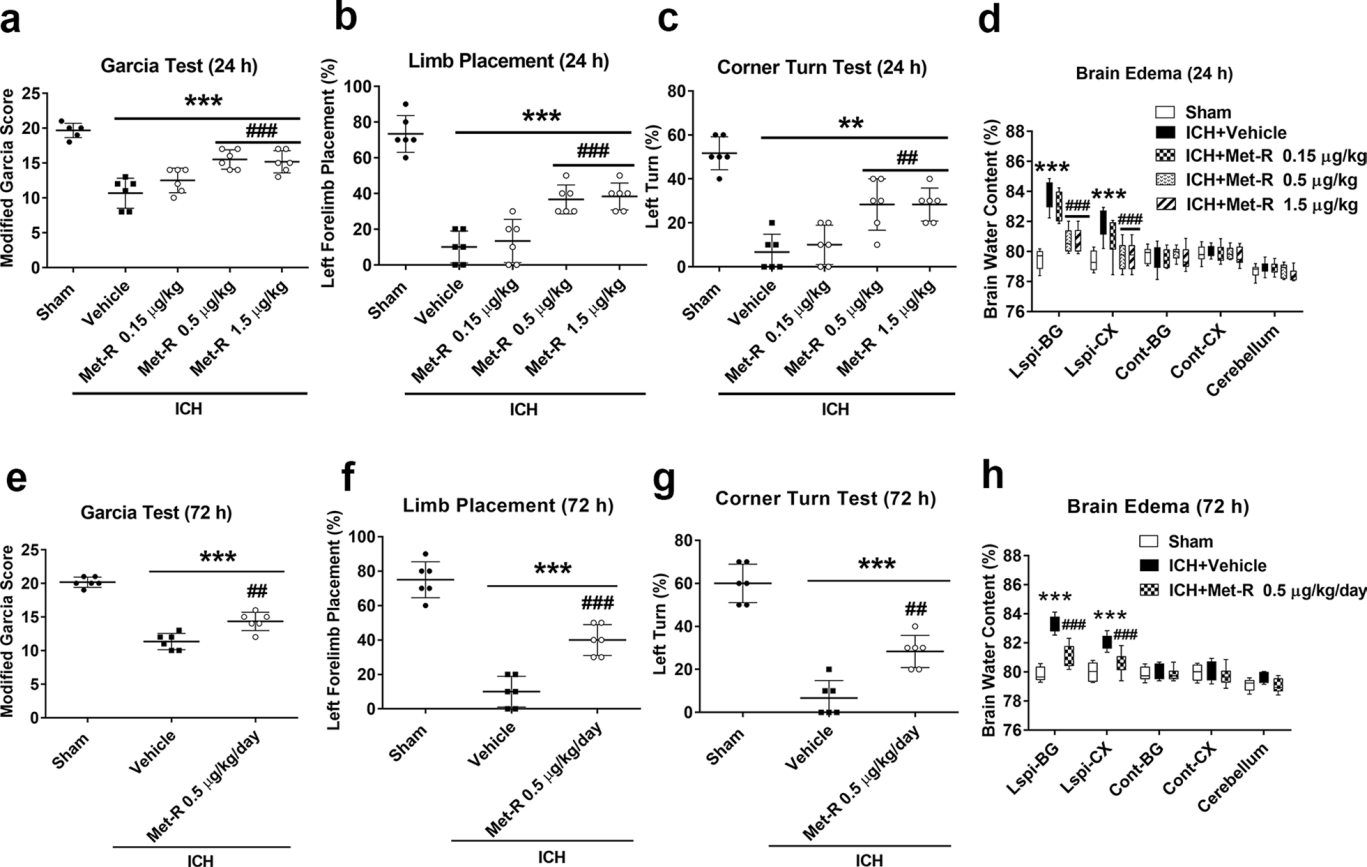
Administration of Met-R decreased brain edema and improved neurological deficits at 24 h and 72 h after ICH in mice. **A** Modified Garcia test, **B** Limb placement test, **C** Corner turn test, and **D** Brain edema at 24 h following surgery in sham, vehicle-treated ICH, and Met-R (0.15, 0.5, and 1.5 μg/kg/day)-treated ICH groups.** E** Modified Garcia test, **F** Limb placement test, **G** Corner turn test and **H** Brain edema at 72 h following surgery in sham, vehicle-treated ICH, and Met-R (0.5 μg/kg/day)-treated ICH groups. Brain sections were divided into five parts: Ipsi-BG (ipsilateral basal ganglia), Cont-BG (contralateral basal ganglia), Ipsi-CX (ipsilateral cortex), Cont-CX (contralateral cortex), and Cerebel (cerebellum). Data were expressed as mean ± SD or median with range. ^**^p < 0.01, ^***^p < 0.001 vs. sham; ^##^*p* < 0.01, ^###^*p* < 0.001 vs. vehicle; n = 6/group

Intranasal Met-R was delivered in three different dosages (0.15, 0.5, and 1.5 μg/kg/day) 1 h post-ICH. Met-R at a dose of 0.5 μg/kg/day significantly improved neurological outcomes in mGarcia, forelimb placement, and corner turn tests 24 h post-ICH (*P* < 0.05; Fig. 3a–c). There was a significant elevation of brain water content in the ipsilateral basal ganglia (ipsi-BG) and the ipsilateral cortex (ipsi-CX) at 24 h after ICH. However, the Met-R (0.5 μg/kg/day) treatment group had significantly less brain water content in both ipsi-BG and ipsi-CX regions compared to the vehicle group 24 h post-ICH (*P* < 0.05; Fig. 3d). Thus, Met-R (0.5 μg/kg/day) was selected as the optimal dose for the following 72 h and long-term outcomes, as well as mechanism studies.

At 72 h post-ICH, the Met-R (0.5 μg/kg/day) treatment significantly improved neurological performance in the mGarcia, forelimb placement, and corner turn tests compared to vehicle group (*P* < 0.05; Fig. 3e–g). The Met-R (0.5 μg/kg/day) treatment group consistently showed significantly less brain water content in both ipsi-BG and ipsi-CX than the vehicle group (*P* < 0.05; Fig. 3h) at 72 h post-ICH.

### Met-R treatment attenuated blood–brain barrier permeability, but not hematoma volume 24 h post-ICH

**Fig. 4:**
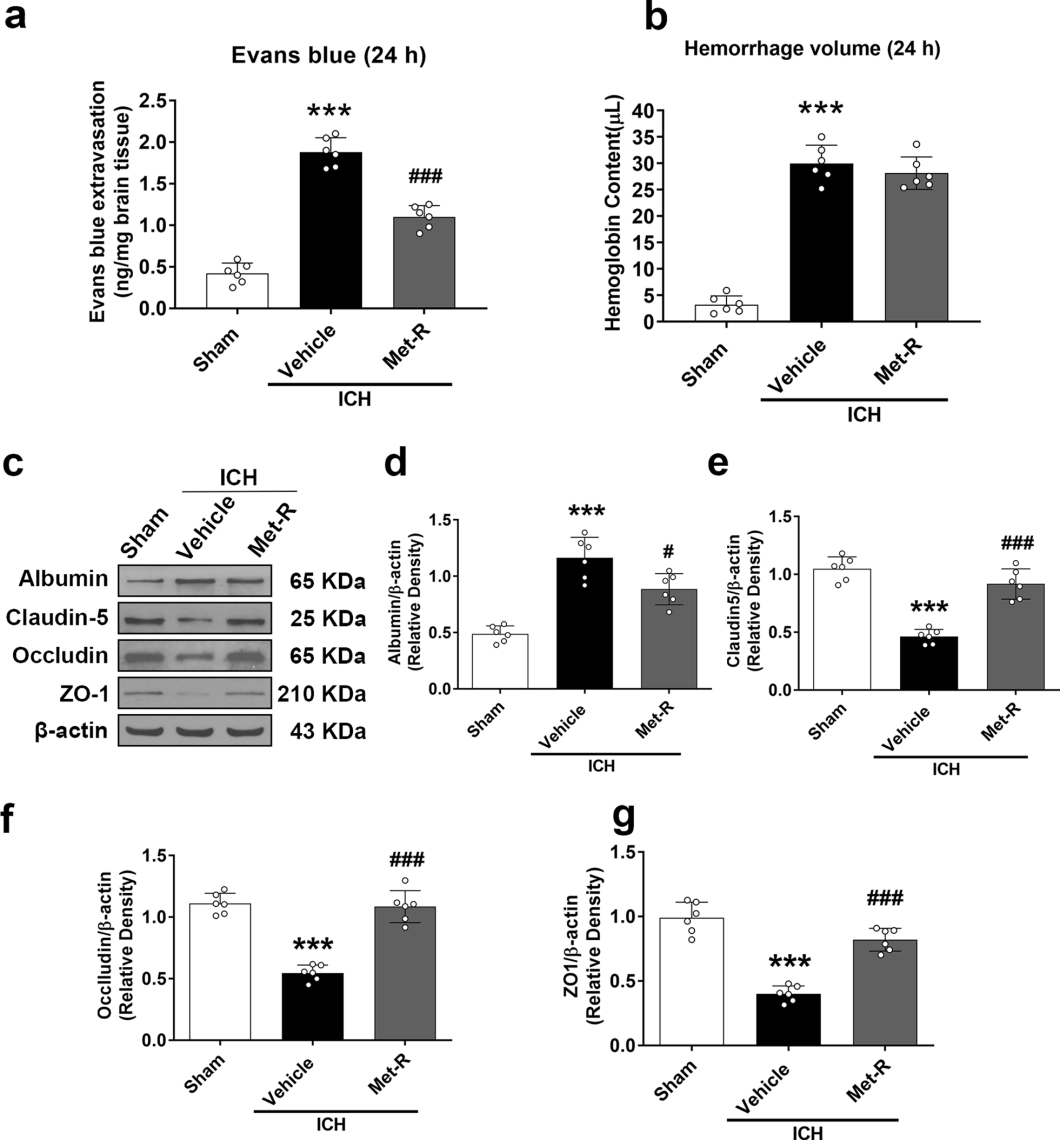
Administration of Met-R preserved blood–brain barrier integrity, but did not affect hemorrhage volume at 24 h after ICH in mice. **A** Evans blue dye extravasation and **B** hemorrhage volume assessment 24 h following surgery in sham, vehicle-treated ICH, and Met-R (0.5 μg/kg/day)-treated ICH groups. **C**, **D**-**G** Representative western blot images and quantitative analyses of albumin, claudin-5, occludin, and ZO-1 in sham, vehicle-treated ICH, and Met-R (0.5 μg/kg/day)-treated ICH groups. Data were expressed as mean ± SD or median with range. ^*^p < 0.05, ^**^p < 0.01, ^***^p < 0.001 vs. sham; ^#^*p* < 0.05 ^##^*p* < 0.01, ^###^*p* < 0.001 vs. vehicle; n = 6/group

Blood brain barrier permeability was assessed in animals from sham, vehicle, and Met-R (0.5 μg/kg/day) treatment groups 24 h post-ICH surgery using Evans blue extravasation assay. The ICH + vehicle group demonstrated a statistically significant increase in Evans blue dye extravasation in comparison to the ipsilateral hemispheric tissues extracted from the sham group (*P* < 0.05; Fig. 4a). However, Met-R (0.5 μg/kg/day) treatment significantly reduced the amount of Evans blue within the brain when compared with the 24 h post-ICH vehicle group.

Hematoma volume was measured using a hemoglobin assay, which revealed that the amount of hemoglobin content was significantly greater in ICH animals compared to the sham group 24 h post-ICH. Met-R (0.5 μg/kg/day) treatment did not reduce hematoma volumes compared to the vehicle group (*P* < 0.05; Fig. 4b). It suggested that the reduction in brain edema and BBB permeability by Met-R treatment was not related to the smaller hematoma size. Western blot analyses showed that albumin expression in the brain tissues of ICH animals was significantly increased, but tight junction protein expression (claudin-5, occludin, and ZO-1) was significantly decreased at 24 h post-ICH compared to the sham group (*P* < 0.05; Fig. 4c–g). The Met-R (0.5 μg/kg/day) treatment group significantly decreased albumin expression, but preserved tight junction protein expression when compared to vehicle group.

### Met-R treatment ameliorated long-term post-ICH neurobehavioral deficits

**Fig. 5:**
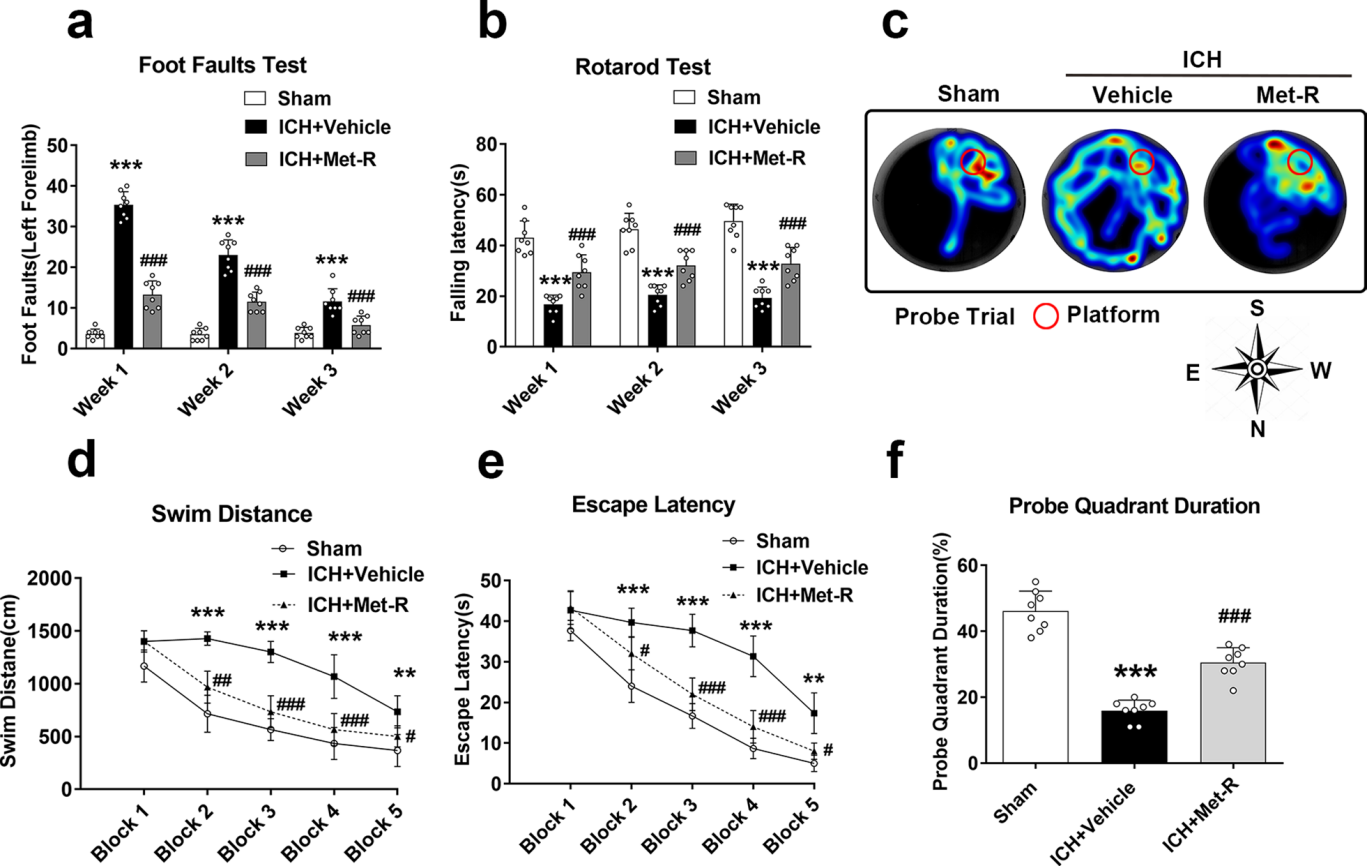
Administration of Met-R ameliorated long-term neurobehavioral deficits 4 weeks after ICH in mice. **A** Foot fault test and **B** Rotarod test. **C** Representative heat map in probe test and **D** swim distance of Morris water maze 21–25 days after ICH. **E** Escape latency and **F** Duration in the probe quadrant. Data were expressed as mean ± SD or median with range. ^*^p < 0.05, ^**^p < 0.01, ^***^p < 0.001 vs. sham; ^#^*p* < 0.05 ^##^*p* < 0.01, ^###^*p* < 0.001 vs. vehicle; n = 8/group

In the foot-fault test, animals in the ICH + vehicle group had more contralateral side (left) foot-faults compared to the shams; the foot faults were significantly improved in the Met-R (0.5 μg/kg/day) treatment group compared to the vehicle group (*P* < 0.05; Fig. 5a). In the rotarod test, the ICH + vehicle group had a significantly reduced falling latency when compared to the sham group; Met-R (0.5 μg/kg/day) treatment markedly improved rotarod falling latency on weeks 1–3 post-ICH when compared to the vehicle group (*P* < 0.05; Fig. 5b).

The Morris water maze test, which evaluates swim distance, escape latency, and probe quadrant duration, was used to assess the effects of Met-R treatment on post-ICH spatial learning capacity and memory function recovery. The animals in ICH + vehicle group had significant neurological dysfunction when compared with the sham group. However, the Met-R treatment group significantly improved memory function and learning ability compared to the vehicle group as deduced from the reduced swimming distance (*P* < 0.05; Fig. 5d) and time to find platform(*P* < 0.05; Fig. 5e), but with more time spent in platform quadrant (*P* < 0.05; Fig. 5c, f).

### Rac1 CRISPR Activator (CRISPRa) reversed Met-R’ neuroprotective effects, as well as CCR1/SRC/Rac1-Mediated BBB permeability 24 H Post-ICH

**Fig. 6:**
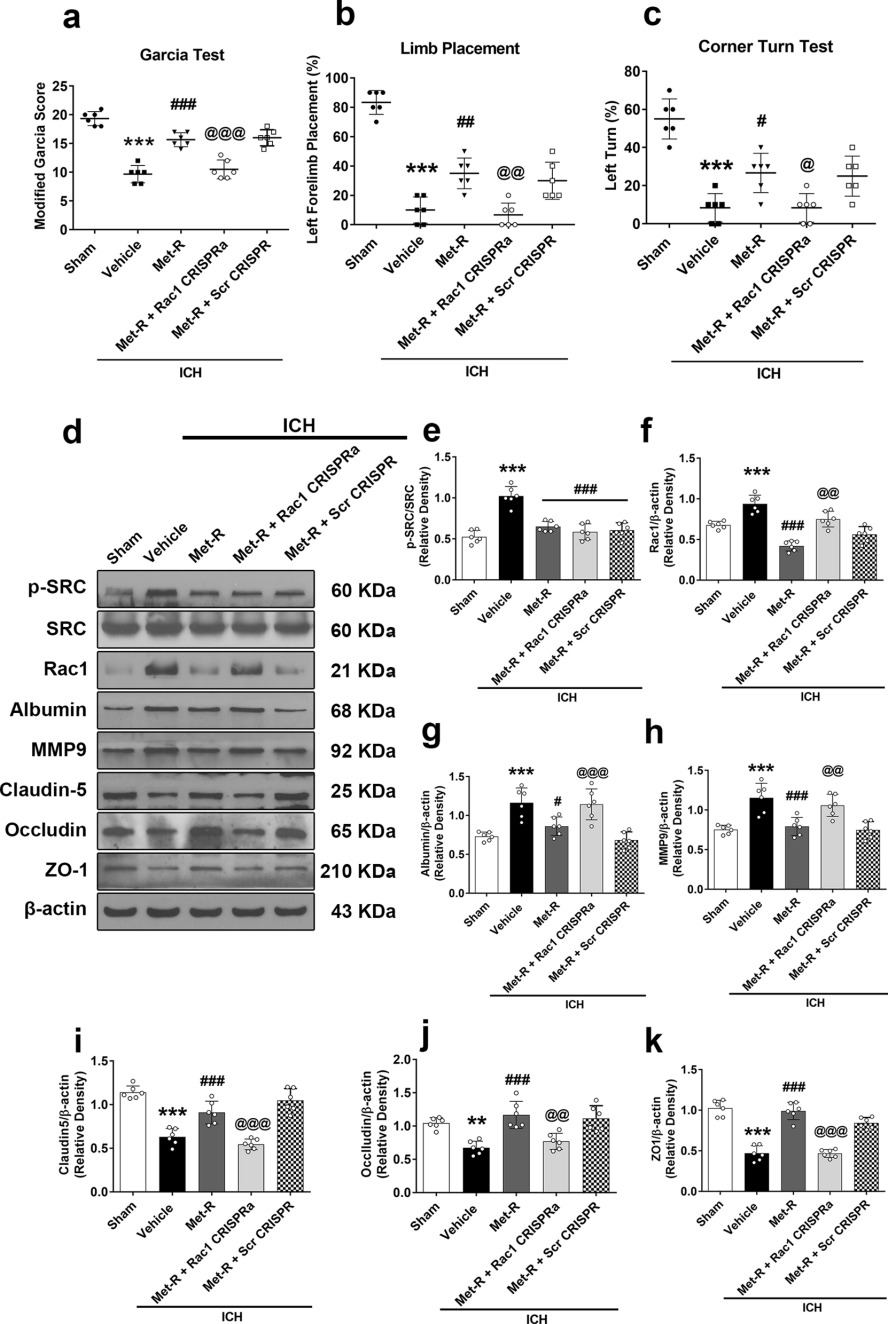
Activation of Rac1 with Rac1 CRISPR Activation (CRISPRa) abolished the beneficial effects of Met-R on neurological deficits and BBB disruption 24 h after ICH mice. **A** Modified Garcia test. **B** Forelimb placement test. **C** Corner turn test. **D** Representative western blot bands. **E**–**K** Quantitative analyses of CCR1, p-SRC, Rac1, albumin, MMP9, claudin-5, occludin, and ZO-1 in the ipsilateral hemisphere at 24 h after ICH. ^*^p < 0.05, ^**^p < 0.01, ^***^p < 0.001 vs. sham, ^#^*p* < 0.05 ^##^*p* < 0.01, ^###^*p* < 0.001 vs. ICH + vehicle, and ^@^*p* < 0.05 ^@@^*p* < 0.01, ^@@@^*p* < 0.001 vs. ICH + Met-R + Scr CRISPR. Data were expressed as mean ± SD. n = 6 per group

Pretreatment with Rac1 CRISPRa significantly reversed the neurobehavioral benefits of Met-R on the modified Garcia, forelimb placement, and corner turn tests 24 h post-ICH (*P* < 0.05; Fig. 6a–c). Met-R significantly decreased brain p-SRC, Rac1, albumin, and mmp9 protein levels. However, there were increased tight junction proteins including claudin-5, occludin, and ZO-1 within the ipsilateral hemisphere brain tissues post-ICH. The Rac1 CRISPRa consistently reversed the protective effects of Met-R against CCR1/SRC/Rac1-mediated BBB disruption, leading to reduced protein levels of claudin-5, occludin, and ZO-1 compared to the Met-R treatment group (*P* < 0.05; Fig. 6d–k).

### The rCCL5-mediated activation of CCR1 resulted in neurofunctional deficits and decreased BBB permeability in naïve animals

**Fig. 7:**
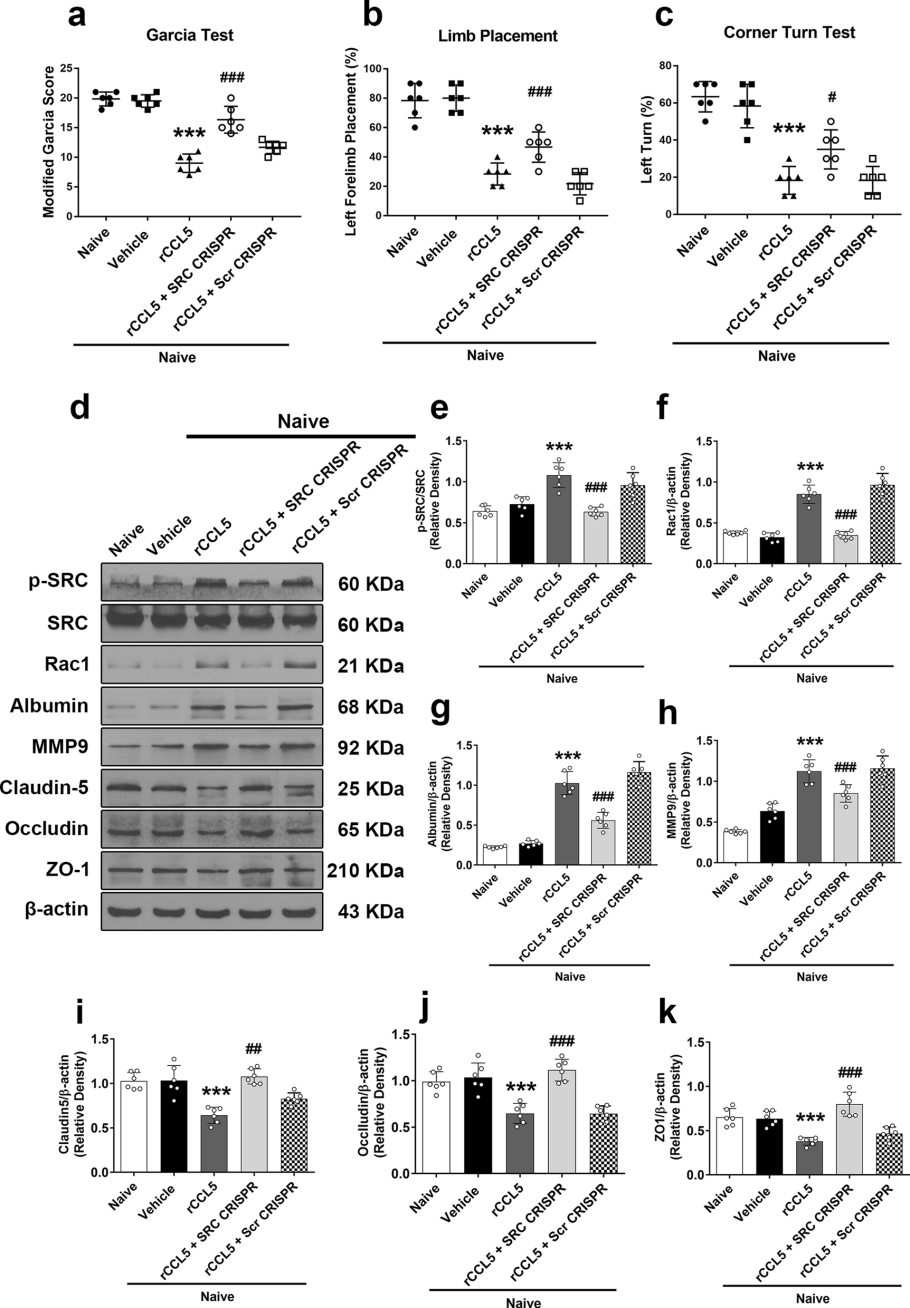
The effects of rCCL5 and SRC CRISPR (KO) on neurological outcomes and expression of CCR1 and its downstream signaling proteins in naïve mice. **A** Modified Garcia test. **B** Forelimb placement test. **C** Corner turn test. **D** Representative western blot bands. **E**–**K** Quantitative analyses of CCR1, p-SRC, Rac1, albumin, MMP9, claudin-5, occludin, and ZO-1 in the ipsilateral hemisphere 24 h post-ICH. ^*^p < 0.05, ^**^p < 0.01, ^***^p < 0.001 vs. Naïve, ^#^*p* < 0.05 ^##^*p* < 0.01, ^###^*p* < 0.001 vs. Naïve + vehicle. Data were expressed as mean ± SD. n = 6/group

Intracerebroventricular injection of rCCL5, a CCR1 ligand, significantly reduced neurological scores of the modified Garcia, forelimb placement, and corner turn tests in naïve animals (*P* < 0.05; Fig. 7a–c), which were associated with significantly higher p-SRC, Rac1, albumin, and MMP9 protein levels in brain tissue. There were decreased tight junction proteins, including ZO-1, occludin, and claudin-5, in the ipsilateral hemisphere of the brain. SRC CRISPR knockout, but not Scramble CRISPR, reversed the detrimental effects of rCCL5 by significantly reducing SRC expression (*P* < 0.05; Fig. 7d–k). The results suggested that activation of CCR1/SRC/Rac1 signaling pathway increased BBB permeability.

## Discussion

ICH is a devastating subtype of stroke with a current lack of effective treatments. An intact BBB is a natural barrier to the central nervous system and provides an ideal environment that is vital to preserving neurological function [38]. Endothelial cells, interconnecting via tight junction proteins, are the primary component of the BBB. Breakdown of these tight junction proteins (occludin, claudin, and ZO-1) directly impact the structural stability of the BBB [39]. Therefore, tight junction protection may minimize BBB impairment, thus promoting neurological functional recovery, decreasing post-ICH SBI.

In the current study, using a mouse model of ICH, the role of CCL5/CCR1 signaling pathway in BBB disruption was further explored. Treatment with the selective CCR1 antagonist, Met-R, demonstrated a protective effect on BBB permeability in ICH mice. Our results showed that (1) CCL5, CCR1, p-SRC, and Rac1 protein levels were increased in ipsilateral brain tissue after ICH, peaking at 24 h post-ICH. CCR1 expression was co-localized with endothelial cells, astrocytes, and neurons; (2) CCR1 inhibition with Met-R after ICH improved short- and long-term neurological deficits, reduced cerebral edema and decreased the expression of CCR1, CCL5, p-SRC, Rac1, albumin, and MMP9, but increased expression of claudin-5, occludin, and ZO-1 at 24 h post-ICH. Conversely, the Rac1 CRISPR activator reversed the benefits of Met-R; (3) Brain CCR1 activation via i.c.v. administration of rCCL5 induced neurological and BBB impairments in naïve mice, whose detrimental effects were attenuated by suppressing brain SRC expression using SRC CRISPR in prior. In conjunction, our findings suggest that cerebral CCR1 activation contributed to post-ICH BBB impairment, partly through the CCR1/SRC/Rac1 signaling pathways. Selective inhibition of CCR1 with Met-R exerted neuroprotective effects on BBB integrity and improved neurological outcomes in ICH mice.

Chemokines, described as small cytokines or basic signaling proteins released by cellular secretion, are responsible for regulating the activation and migration of target cells [40]. These chemokines are categorized as four main subfamilies: CXC, CC, CX3C, and XC according to their behavioral, structural, functional, and genetic characteristics. The ligands of CCR1 include CCL3, CCL5, CCL7, and CCL23. However, CCL5 was the most sensitive and effective independent predictor compared with other ligands in a variety of disease models [41, 42]. Consistent with the above, we found that the expression of CCL5 protein was elevated in the perihematomal zone after ICH in mice. Moreover, increased CCL5 expression was associated with the degree of perihematomal edema.

CCR1 is reportedly expressed on microvascular endothelial cells, vascular smooth muscle cells, astrocytes, and neurons [8–10]. CCR1, activated by its endogenous ligand, CCL5, contributes to the BBB damage in a variety of neuroinflammatory diseases [11, 12]. A previous study indicated that CCR1 mRNA and protein expression were increased in blood plasma, peripheral lymphoid organs, spinal cords, and brains from mice 22 h after middle cerebral artery occlusion (MCAO) [43]. Consistent with the above, our immunofluorescence staining results demonstrated that CCR1 was expressed on endothelial cells, astrocytes, and neurons after ICH. CCR1 expression was increased in a time-dependent manner in the ipsilateral/right hemisphere after ICH. Increasing evidence indicates that CCL5 binds with CCR1 to inhibit tissue recovery and promote inflammation in epithelial cells [44, 45]. We further assessed the effect of the CCL5/CCR1 signaling pathway regarding post-ICH BBB protection.

Met-R is a selective CCR1 antagonist that is effective in treating immune-mediated acute and chronic models of tissue inflammation in vivo [17, 18]. Numerous studies have revealed the anti-inflammatory effects of Met-R, which functions by inhibiting CCR1, and the subsequent suppression of proinflammatory cytokine released in several diseases, including multiple sclerosis (MS), autoimmune encephalomyelitis (EAE) [26], atherosis [46], arthritis [47], and herpes simplex virus infection [48]. Our study is the first to utilize Met-R as a protective agent in maintaining post-ICH BBB integrity. Cerebral edema is a pathological phenomenon associated with BBB impairment and inflammation, resulting in neurologic deficits after ICH [49]. Three different doses of Met-R post-treatment groups (0.15, 0.5, and 1.5 μg/kg/day) were tested in the mice model of ICH. Our present study found that cerebral edema was decreased with three different doses of Met-R 24 h post-ICH. However, the dose of 0.5 μg/kg/day had the highest treatment efficacy in attenuating brain edema and neurobehavioral deficits 24 h after ICH. Therefore, we used the dose of Met-R (0.5 μg/kg/day) for all other experiments in our study. Consistently, Met-R, at a dose of 0.5 μg/kg/day, promoted neurological functional recovery and decreased cerebral edema 72 h post-ICH. The early protection led to improved long-term post-ICH spatial learning deficits and movement discoordination. Associated with neurobehavioral benefits, Met-R exhibited protective effects on BBB integrity after ICH, as evidenced by reduced albumin expression and Evans blue dye extravasation in brain parenchyma, as well as the preservation of tight junction protein expression after ICH in mice.

Next, we assessed the downstream signaling of BBB protection underlying Met-R-mediated CCR1 inhibition. Endothelial SRC phosphorylation could lead to the degradation of transmembrane protein vascular endothelial‐cadherin (VE‐cadherin), as well as the breakdown of blood‐brain barrier (BBB) integrity, thus inducing brain edema after ICH through the PAR1/p-SRC/p-PAK1 signaling pathway in a rat model of hydrocephalus [50]. SRC phosphorylation activates Rac1 proteins responsive to tissue factor-induced cellular apoptosis in endothelial cells [51]. Western blot results in our study showed that Met-R treatment downregulated the expression of p-SRC, Rac1, albumin, and MMP9, but upregulated the expression of claudin-5, occludin, and ZO-1 after ICH. Application of Rac1 CRISPR activator before ICH significantly increased Rac1 expression in brain samples, but reversed the beneficial effects of Met-R on neurobehavioral impairment and BBB breakdown after ICH. These findings suggest that Rac1 might be the downstream signaling factor of CCR1 activation in impairing BBB integrity after ICH.

**Fig. 8:**
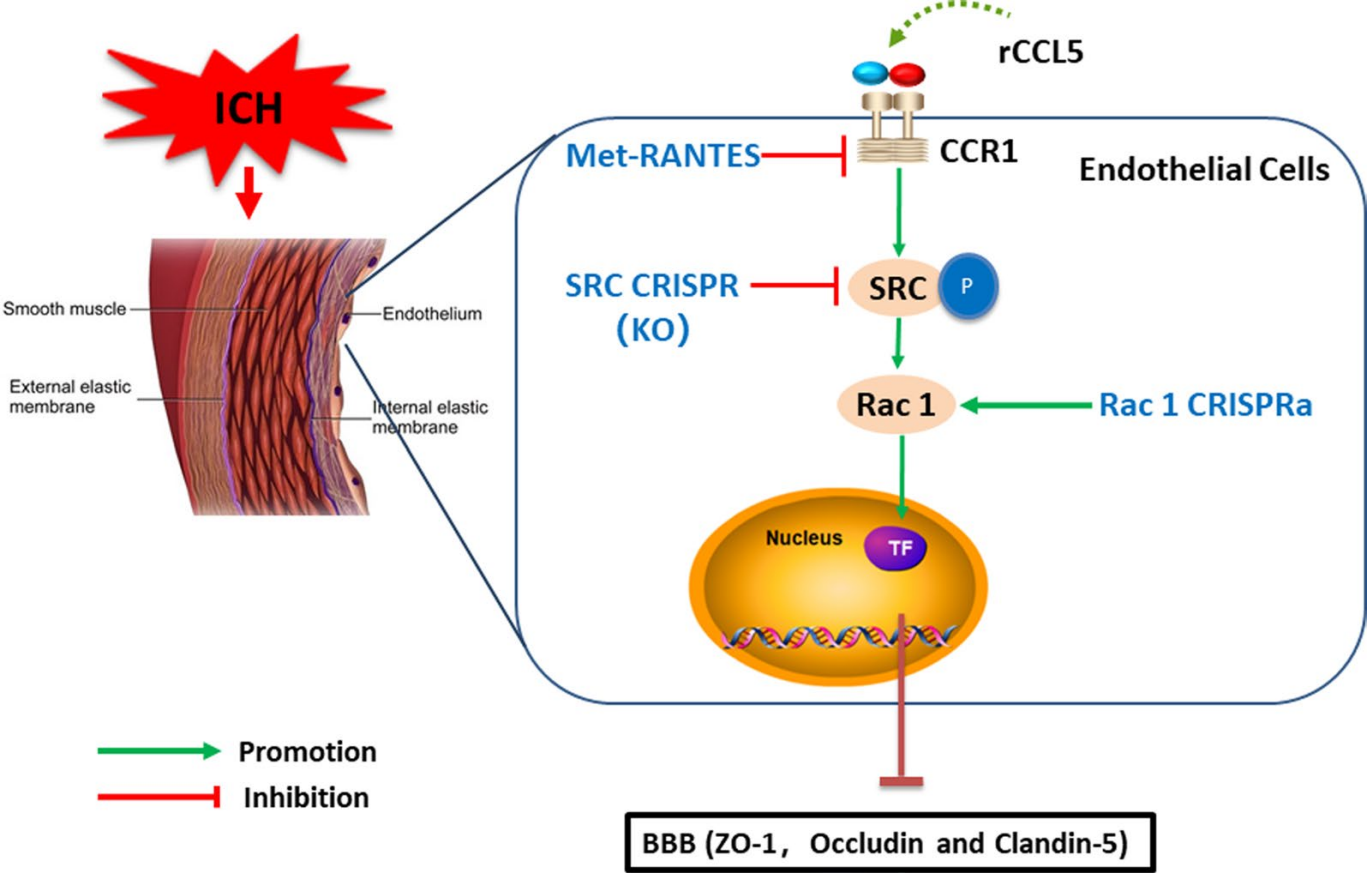
Diagram showed that Met-R preserves the blood–brain barrier integrity after ICH in mice, partially exerting effects through inhibition of CCR1/SRC/Rac1 pathway

Given that Rac is downstream of SRC, the Rac1 CRISPR activator did not alter Met-R-mediated downregulation of SRC in brain tissue after ICH. SRC is a membrane-associated, nonreceptor tyrosine kinase [19]. Although the biological function of SRC is not currently well-defined, it has been shown to contribute to epithelial cell adhesion and motility [20]. Recent studies have revealed that CCR7 triggers an endomembrane signaling complex for spatial Rac activation through the SRC/Rac1 signaling pathway in dendritic cells [23]. For further validation of this pathway, CCR1 activation in the brain of naïve animals, through intracerebroventricular administration of rCCL5, promoted expression of p-SRC, Rac1, albumin, and MMP9, but downregulated the expression of tight junctions including claudin-5, occludin, and ZO-1, leading to neurobehavioral impairments. However, the detrimental effects of CCR1 activation were reversed by SRC CRISPR KO, which attenuated brain edema and ameliorated neurological dysfunction. Therefore, our findings support the hypothesis that Met-R-mediated CCR1 inactivation may preserve BBB integrity, possibly in part through inhibiting the CCR1/SRC/Rac1 signaling pathway (Fig. 8).

There are several limitations to this study. First, the current emphasis of this study lies in uncovering the role of CCR1 in preserving post-ICH BBB integrity. Our previous research has shown that CCR1 mediated neuroinflammation after ICH [25]. Thus, we cannot exclude that the anti-inflammatory effects provided by Met-R contributed to the neurobehavioral benefits. Second, ICH-induced impairment of the BBB is a complex pathological process. Given the essential role of Src-Rac1 signaling pathway in cell proliferation [52], the effects of Met-R treatment on inhibitions of neurogenesis and self-repair in chronic phase after ICH need to be further elucidated. Third, in the absence of gelatin zymography evaluation, MMP9 changes examined by western blot did not provide information regarding MMP9 activation/function in our study. Fourth, only male animals were used in this study. Sexual dimorphism has been implicated as a possible variable which could affect BBB disruption. Previous studies have suggested that female mice have lower mortality and better neurobehavioral prognosis compared to male mice following ICH [53]. Treatment effects of Met-R in female animals after ICH should be elucidated in future studies.

## Conclusions

Our study suggests that CCR1 activation aggravated BBB disruption, neurological impairments, and brain edema in mice after ICH. CCR1 inhibition with Met-R preserved BBB integrity at least in part through inhibiting CCR1/SRC/Rac1signaling pathway. Thus, Met-R has therapeutic potential in the management of ICH-induced BBB impairments in patients.

## Supplementary Information

**Additional file 1: Table S1.** Animal Groups and Number of Mice Used in the Study.

## Data Availability

The datasets analyzed during the current study are available from the corresponding author on reasonable request.

## Abbreviations

BBB: Blood brain barrier
ARRIVE: Animal Research: Reporting of In Vivo Experiments
CCR1: C–C chemokine receptor type 1
Cerebel: Cerebellum
CNS: Central nervous system
Cont-BG: Contralateral basal ganglia
Cont-CX: Contralateral cortex
GFAP: Glial fibrillary acidic protein
ICH: Intracerebral hemorrhage
Ipsi-BG: Ipsilateral basal ganglia
Ipsi-CX: Ipsilateral cortex
Rac1: Ras-related C3 botulinum toxin substrate 1
Met-R: Met-RANTES
NeuN: Neuronal specific nuclear protein
PBS: Phosphate-buffered saline
