# Supplementary material for: Met-RANTES preserves the blood–brain barrier through inhibiting CCR1/SRC/Rac1 pathway after intracerebral hemorrhage in mice
Source: Fluids Barriers CNS. 2022 Jan 21;19:7. doi: 10.1186/s12987-022-00305-3 (PMC8781527; doi:10.1186/s12987-022-00305-3)
Supplement: Supplementary file 1 — Additional file 1: Table S1. Animal Groups and Number of Mice Used in the Study. [file 12987_2022_305_MOESM1_ESM.docx]

**Table S1.** Summary of experimental groups and mortality rate in the study.

| **Experimental Groups** | **Neurological test** | **IHC** | **WB** | **Hemorrhage volume** | **Exclusion** | **Mortality** | **Subtotal** |
| --- | --- | --- | --- | --- | --- | --- | --- |
|  | **Brain water content** |  |  | **Evans Blue** |  | **(%)** |  |
| **Experimental 1** |  |  |  |  |  |  |  |
| Sham |  | 2 | 6 |  | 0 | 0 | 8 |
| ICH (3h, 6h, 12h, 24h, 72h) |  | 2 | 30 |  | 0 | 2(5.56%) | 34 |
| **Experimental 2** |  |  |  |  |  |  |  |
| Sham | 12 | 4 | 6 |  | 0 | 0 | 22 |
| ICH + Vehicle | 12 | 4 | 6 |  | 0 | 0 | 22 |
| ICH + Met-R 0.15 μg/kg/day | 6 |  |  |  | 0 | 1(14.29%) | 7 |
| ICH + Met-R 0.5 μg/kg/day | 12 | 4 | 6 |  | 0 | 1(4.35%) | 23 |
| ICH + Met-R 1.5 μg/kg/day | 6 |  |  |  | 0 | 0 | 6 |
| **Experimental 3** |  |  |  |  |  |  |  |
| Sham | 8 |  |  |  | 0 | 0 | 8 |
| ICH + Vehicle | 8 |  |  |  | 0 | 1(11.11%) | 9 |
| ICH + Met-R | 8 |  |  |  | 0 | 1(11.11%) | 9 |
| **Experimental 4** |  |  |  |  |  |  |  |
| Sham |  |  |  | 6 | 0 | 0 | 6 |
| ICH + Vehicle |  |  |  | 6 | 0 | 1(14.29%) | 7 |
| ICH + Met-R |  |  |  | 6 | 0 | 1(14.29%) | 7 |
| Sham + Evans Blue |  |  |  | 6 | 0 | 0 | 6 |
| ICH + Vehicle + Evans Blue |  |  |  | 6 | 0 | 1(14.29%) | 7 |
| ICH + Met-R + Evans Blue |  |  |  | 6 | 0 | 1(14.29%) | 7 |
| **Experimental 5** |  |  |  |  |  |  |  |
| Sham |  |  | 6 |  | 0 | 0 | 6 |
| ICH + Vehicle |  |  | 6 |  | 0 | 0 | 6 |
| ICH + Met-R |  |  | 6 |  | 0 | 1(14.29%) | 7 |
| ICH + Met-R + Rac1 CRISPRa |  |  | 6 |  | 0 | 1(14.29%) | 7 |
| ICH + Met-R + Scr CRISPR |  |  | 6 |  | 0 | 0 | 6 |
| **Experimental 6** |  |  |  |  |  |  |  |
| Naive |  |  | 6 |  | 0 | 0 | 6 |
| Naive + Vehicle |  |  | 6 |  | 0 | 0 | 6 |
| Naive + rCCL5 |  |  | 6 |  | 0 | 0 | 6 |
| Naive + rCCL5 + SRC CRISPR |  |  | 6 |  | 0 | 0 | 6 |
| Naive + rCCL5 + Scr CRISPR |  |  | 6 |  | 0 | 0 | 6 |
| **Total** | 72 | 16 | 114 | 36 | 0 | 12(4.8%) | 250 |

ICH, intracerebral hemorrhage; WB, western blot; IHC, immunohistochemistry.
